# Therapeutic exercise interventions in pediatric survivors of brain cancer and other solid tumors: A scoping review

**DOI:** 10.3389/fped.2022.979292

**Published:** 2022-09-16

**Authors:** Brooke E. Kohler, Carolina X. Sandler, Emmah Baque, Natalie K. Bradford, Stewart G. Trost

**Affiliations:** ^1^Faculty of Health at the Queensland Centre for Children's Health Research, Queensland University of Technology (QUT), Brisbane, QLD, Australia; ^2^Sport and Exercise Science, School of Health Sciences, Western Sydney University, Sydney, NSW, Australia; ^3^UNSW Fatigue Research Program, Kirby Institute, University of New South Wales, Sydney, NSW, Australia; ^4^Menzies Health Institute Queensland, Griffith University, Brisbane, QLD, Australia; ^5^School of Health Sciences and Social Work, Griffith University, Nathan, QLD, Australia; ^6^Cancer and Palliative Care Outcomes Centre, at Centre for Children's Health Research, Queensland University of Technology, South Brisbane, QLD, Australia; ^7^School of Human Movement and Nutrition Sciences, University of Queensland, Brisbane, QLD, Australia

**Keywords:** children, pediatrics, oncology, solid tumor, physical activity, therapy, survivorship

## Abstract

**Background:**

Improved survival rates for children with solid tumors presents an ongoing challenge of how to maximize quality of survivorship and effectively manage the short- and long-term complications of disease and treatment. To gain an understanding of the extent and nature of research pertaining to therapeutic exercise interventions and identify knowledge gaps, we conducted a scoping review of exercise training studies conducted in pediatric survivors of brain cancer and other solid tumors.

**Method:**

A systematic literature search was performed across four electronic databases. Papers were selected for full-text review if they included participants treated for brain cancer or other solid tumors, with at least 50% of participants aged ≤ 21 years, evaluated an exercise intervention ≥2-weeks in duration, and were published in an English, peer-reviewed journal. We included the following quantitative study designs; randomized controlled trials, non-randomized trials, and single-arm pre-test-post-test.

**Results:**

Of the 7,482 citations identified, 17 papers met the inclusion criteria (presenting findings from eleven studies). Two studies were randomized controlled trials, five studies were non-randomized controlled trials, and four studies were a single-arm pre-test post-test design. Average age of participants ranged from 7.3–15.5 years, and time since diagnosis ranged from 3 to 70 months. Five studies included participants with brain tumors exclusively, three studies included other solid tumors, and three studies included a mixed sample (brain and other solid tumors). A wide range of exercise modalities were employed, including cycle ergometry, resistance training, sport, yoga, and active gaming. The length of the exercise program ranged from 3–40 weeks and frequency from 3–11 sessions per week. Exercise session duration ranged from 15–180 min, with most studies reporting 30–90-min sessions. Adherence ranged from 77 to 100%, with none of the studies reporting adverse events. Studies reported improvements in cardiorespiratory fitness, functional strength, physical activity, and quality of life.

**Conclusions:**

A small number of mostly low methodological quality studies have examined the effects of therapeutic exercise in pediatric survivors of solid tumors. Although limited, the extant literature supports the feasibility and safety of therapeutic exercise interventions for pediatric survivors of brain cancer and other solid tumors.

## Introduction

Solid tumors account for approximately 40–60% of cancer diagnoses in children and adolescents (aged 0–21 years) worldwide (1–3). Solid tumor types include tumors of the central nervous system, neuroblastomas, Wilms tumor, Ewing's tumor, rhabdomyosarcoma, soft tissue sarcomas, germ cell tumor and melanomas, and unlike adult cancers, childhood cancers are characterized by their cell of origin, not by their location (4). With advances in surgical intervention, radiotherapy, chemotherapy, and stem cell transplantation, the survival rates of children with solid tumors have increased dramatically over the past several decades, with the current overall 5-year survival rates ranging from 74 to 99% (2, 3). Improved survival rates present an ongoing challenge in survivorship of how to effectively manage the short- and long-term complications acquired from the disease and treatment. Further, the prevention of disabling secondary chronic health conditions, such as cardiovascular disease, metabolic disorders and secondary malignancies is an important factor of care post treatment. In this review, a “survivor” is considered any individual diagnosed and treated for cancer (5).

Pediatric survivors of solid tumors experience a myriad of short- and long-term complications following treatment, as well as late effects following treatment. Common late effects include reduced strength, poor cardiorespiratory fitness, increased fatigue, lowered executive function, and increased pain, consequently making home, school, and recreation activities more challenging (6–11). Quality of life in survivorship is a major concern for patients, families, and clinicians, with survivors of childhood solid tumors reporting a more severe impact of cancer and treatment into adulthood, compared to other cancers (12, 13). With more children than ever surviving solid tumors and living with late effects from the disease and its treatment, there is an urgent need for effective therapies to improve patient outcomes. Physical activity is beneficial for outcomes in both healthy and disease-burdened populations (14). Previous reviews have summarized the research literature on exercise training for mixed pediatric cancer diagnoses (15–18), the combination of adolescent and young adult cancer groups (19, 20), and exercise interventions during the treatment phase (21–23). The results indicate that therapeutic exercise training of sufficient frequency, intensity, and duration can improve cardiorespiratory fitness, muscular strength, fatigue and cognitive functioning (15–17). Additional benefits include improved immune function, reduced days of hospitalization and reduced risk of infection (22). However, the bulk of studies included in these reviews have been conducted in survivors of blood cancers and there is a dearth of research evidence on the efficacy of therapeutic exercise training among pediatric survivors of solid tumors (16, 24). Children with solid tumors differ in their clinical presentation, can receive more intensive treatment combinations of surgery, radiotherapy, and chemotherapy, and thus are likely to respond differently to therapeutic exercise (25–27). To gain a better understanding of the extent and nature of research pertaining to therapeutic exercise programs we conducted a scoping review of exercise training studies conducted in pediatric survivors of brain cancer and other solid tumors.

## Methods

### Search strategy

A systematic literature search was performed across four electronic databases: Embase, CINAHL, PubMed and Scopus in June 2020, and updated in April 2022. Search terms were developed in consultation with a librarian and based on previously conducted systematic reviews (18, 20, 28). Search terms were deliberately kept broad to ensure the full scope of the research was identified. No limits on publication date were applied. The search strategy contained four key topics: (1) pediatrics and adolescents, (2) solid tumors, (3) physical activity or exercise, and (4) study design (e.g., randomized controlled trials). Details of the search strategies can be found in the Supplementary material 1. The review is reported in accordance with the Preferred Reporting Items for Systematic reviews and Meta-Analyses extension for Scoping Reviews (PRISMA-ScR) (29).

### Inclusion and exclusion criteria

Papers were selected for full text review based on the following criteria: (1) included participants were treated for a solid tumor including tumors of the central nervous system, neuroblastomas, retinoblastomas, renal tumors, hepatic tumors, malignant bone tumors, soft tissue sarcomas, germ cell tumor and/or melanomas; (2) results were reported separately for solid tumors where studies included blood cancers; (3) at least 50% of participants were aged ≤ 21 years; (4) evaluated the effects of a physical activity or exercise intervention with a minimum duration of 2 weeks; and (5) published in an English-language, peer-reviewed journal. Papers were excluded if they evaluated an exercise program which was delivered in combination with other therapies (e.g., cognitive behavioral therapy, diet, and nutrition interventions) or if the record was a conference abstract, unpublished theses, commentary, newsletter, protocol, or case study.

### Selection of included papers

Search results were exported into EndNote (Version X9), duplicates were removed, and citations (title and abstract) uploaded to online systematic review software (Covidence). Each citation was screened against the inclusion and exclusion criteria by at least two independent reviewers (BK, and either EB or CS) in two stages: (1) title and abstract screening and (2) full-text screening. Discrepancies were resolved by a third reviewer (EB or CS). In addition, a hand search of references lists of included papers was undertaken.

### Data extraction

A data extraction table was developed by all authors to collate relevant information about the study design, sample size, tumor type(s), exercise training (location, supervision, frequency, intensity, duration and modes), and reported outcomes. Data extraction for one paper were completed collectively by all authors before one author (BK) completed data extraction for all remaining studies. Methodological quality was assessed independently by two authors (BK, and either EB or CS) using the Physiotherapy Evidence Database Scale (PEDro) (30). Any discrepancies were resolved through consensus. Scores of <4 were considered “poor”, 4 to 5 were considered “fair”, 6 to 8 were considered “good” and 9 to 10 were considered “excellent” (31).

## Results

### Search results

**Figure 1:**
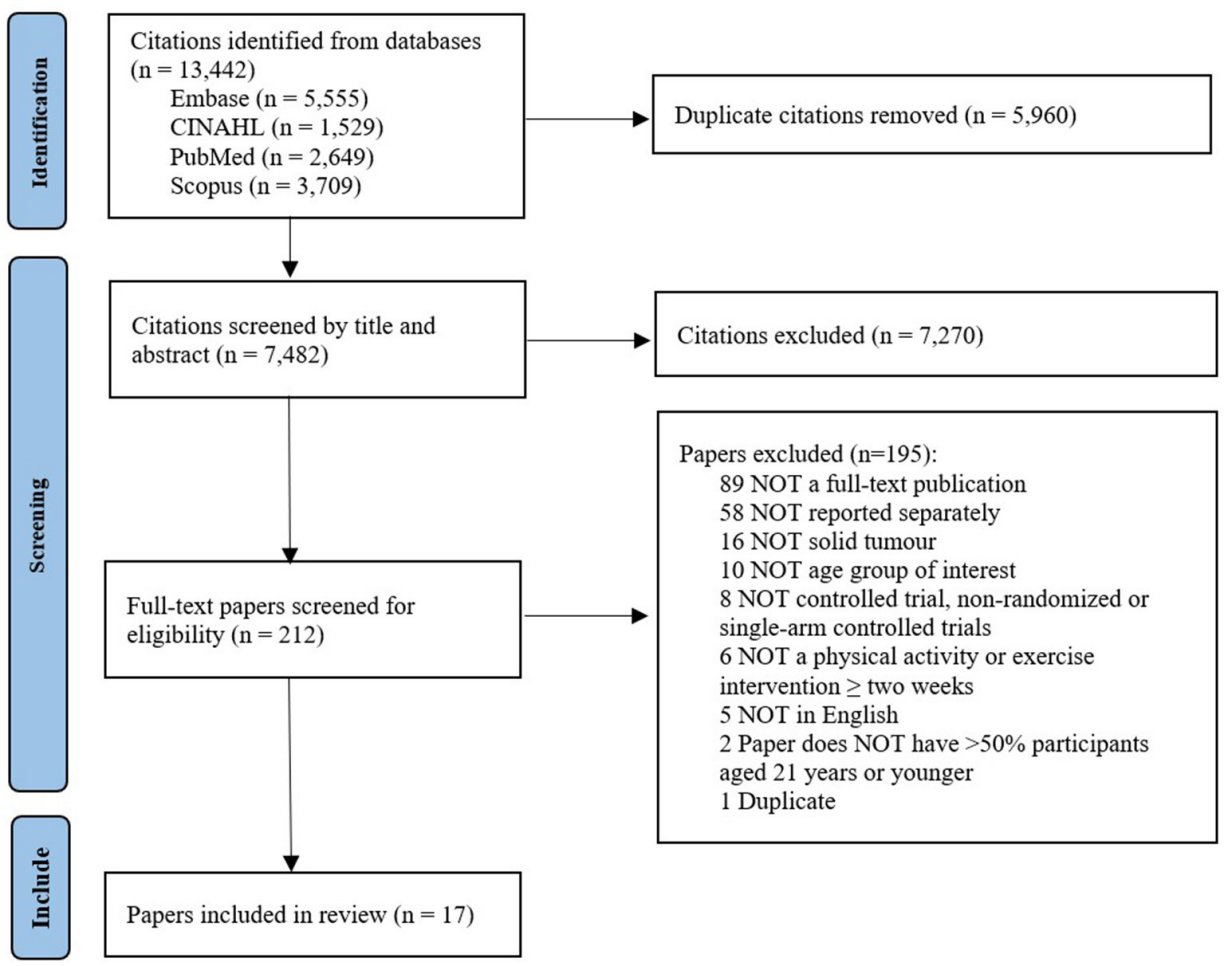
Flow chart of included and excluded papers.

After removal of duplicates, the search identified a total of 7,482 papers. Following title and abstract screening, 212 were selected for full-text review. Of this number, 17 papers presenting findings from 11 studies met the eligibility criteria and were included for data extraction (see Figure 1).

### Study characteristics

Supplementary material 2 provides a detailed summary of the 17 papers. All papers were published between 2014 and 2021. Five of the eleven studies were conducted in North America (32–39), five conducted in Europe (40–47), and one conducted in Asia (48). Two studies were randomized controlled trials (40–42, 47), five studies were non-randomized trials with a control condition (32–36, 39, 45, 46), and four studies were single-arm pre-test post-test studies (37, 38, 43, 44, 48). Five studies conducted assessments immediately post-intervention only (37, 38, 44, 46, 48), five studies conducted follow-ups at 2–6-months post-intervention (32–36, 39–42, 47), and one study conducted follow-up assessments at 12-months post-intervention (43, 45). In single-arm pre-test post-test studies, the sample size ranged between nine (38) and 88 participants (44). In studies with a control group, total sample size ranged between 13 (41, 42) and 57 (39), with control group sample sizes ranging between seven (41, 42) and 35 (32). The average participant age ranged from 7.3 years (38) to 15.5 years (45). Time since diagnosis ranged from 3 months (45) to 70 months (33–36). Five studies included participants with brain cancer exclusively (33–38, 41, 42, 48), three studies included other solid tumors (32, 39, 40, 47), and three studies included mixed sample with brain cancer and other solid tumors (43–46). Specific tumor types included astrocytoma, medulloblastoma, ependymoma, juvenile pilocytic astrocytoma, craniopharyngioma, germ cell tumor, retinoblastoma, choroid plexus carcinoma, glioma, osteosarcoma, Ewing's sarcoma, chondroblastoma, soft tissue sarcomas, neuroblastoma, and Wilms tumor. Based on the PEDro scoring, four papers were rated as “poor” (37, 39, 43, 44), eight papers as “fair” (33–36, 38, 45, 46, 48), four papers as “good” (32, 41, 42, 47), and one paper as “excellent” (40).

### Exercise interventions

Five studies evaluated exercise programs post-treatment (33–38, 41–44) and six studies evaluated exercise programs during treatment (32, 39, 40, 45–48). Six studies examined the effects of a hospital inpatient program (39, 40, 43–48), two examined combined outpatient and home-based programs (33–36, 38), two examined home-based programs (37, 41, 42), and one study examined the effects of a combined hospital inpatient and outpatient program (32). Nine studies evaluated individual exercise programs delivered one-on-one (32, 37–42, 45–48), with the remaining two studies evaluating programs that combined individual and group-based exercise (33–36, 43, 44). In all eleven studies, the exercise program was supervised or remotely coached by a trained health professional (e.g., nurse, physiotherapist, exercise physiologist or trained exercise leader).

Multiple modes or types of exercise were reported across the eleven studies, including aerobic exercise (cycle ergometry, sports, active games) (32–36, 40, 45–47), resistance training (32, 40, 45–47), stretching (32, 45), yoga (48), and active video gaming (41, 42). One study evaluated the efficacy of physical activity goal setting and self-monitoring with an activity tracker (37). One study employed constraint-induced movement therapy (CIMT), which required participants to wear a removable cast on the unaffected arm and engage in task-specific training using the affected arm (38). One study utilized a staged coaching program during routine clinic visits to identify barriers to physical activity and prescribe physical activity and resources accordingly (39). Seven studies reported utilizing adult-like exercise programs (e.g., treadmills, cycle ergometry, resistance training) and/or competitive games/sports (e.g., basketball, relay running, dodgeball) (32–36, 39, 40, 45–48).

The length of the exercise program ranged from 3 weeks (38) to 40 weeks (45), with four studies opting for 10–12 week intervention periods (32–37, 41, 42). Exercise frequency ranged from three (32–36, 40, 45–48) to 11 (43, 44) sessions per week. Two studies did not report frequency (37, 39). The duration of the exercise program ranged from 15 min (45) to 180 min (38), with most studies reporting 30- to 90-min sessions (32–36, 40–48). Seven studies did not report exercise intensity (37–39, 41–44, 46, 48). Studies that reported intensity prescribed moderate-to-high intensity exercise determined by percentage of peak heart rate (33–36), age-predicted heart rate maximum (32, 40, 47), or Borg ratings of perceived exertion (RPE) (45). Resistance training comprised 1–3 sets of 6–15 repetitions for major muscle groups (32, 40, 45).

Adherence was operationally defined differently across studies. Six studies defined adherence as the number of sessions attended divided by the number prescribed, adherence ranged from 77 to 100% (32–36, 38, 41, 42, 45, 48). One study defined adherence as the number of prescribed exercises completed, where 68% of participants completed >90% of prescribed exercises (40, 47). One study defined adherence as the number of weeks the participant met or exceeded their goal, reported to be 69% (37). Three studies did not report adherence (43, 44, 46). Six of the eleven studies monitored adverse events (32–37, 40, 45, 48), with no adverse events reported.

### Outcomes

A total of nine different outcomes were measured across the 17 papers. The most commonly measured outcomes were physical activity (*n* = 6) (37, 39–41, 43, 45), QoL (*n* = 6) (37–40, 43, 46), motor performance (*n* = 5) (35, 38, 41, 42, 44), cardiorespiratory fitness (*n* = 4) (34, 35, 37, 40), and brain structure and function (*n* = 4) (33, 34, 36, 42). There were multiple measures used for each outcome. Physical activity was measured by wearable devices, including step counters (37) and accelerometers (39, 40, 43–45, 47); and/or by self-report questionnaires, including the Godin-Leisure-Time Exercise Questionnaire (37, 39). Motor performance was measured by the Bruininks-Osterestsky Test of Motor Performance (35, 41), the Assessment of Motor and Process Skills (42), gait analysis (44), balance (44), and three upper limb function measures (38). Cardiorespiratory fitness was measured by the Six-Minute Walk Test (34, 37) and/or by a graded exercise test with spirometry (35, 40). According to the International Classification of Functioning, Disability and Health (ICF), studies assessed impairment- or activity-related outcomes; no specific participation-related outcomes were reported, except for QoL (49, 50).

Exercise programs that involved a combination of aerobic and resistance training (*n* = 5) (32, 40, 43–47) resulted in improvements in strength (40), functional mobility (32, 40), physical activity (43, 45), QoL (43), balance (44), immune function (47) and bone mass (45). Exercise programs that involved aerobic training only (*n* = 1) (33–36) resulted in improvements in cardiorespiratory fitness (34, 35), brain structure and function (33, 34, 36), and motor performance (35, 41). Active gaming (*n* = 1) (41, 42) resulted in improvements in physical activity (41), cognitive function (42), coordination (41), and activities of daily living to a score above the cut-off for independent living (42). Goal setting and self-monitoring daily step counts (*n* = 1) (37) resulted in improvements in cardiorespiratory fitness, QoL and fatigue. CIMT (*n* = 1) (38) resulted in improvements in the amount and quality of hemiplegic arm use, general fatigue, and sleep/rest fatigue sub-domain scores. Yoga (*n* = 1) (48) resulted in improvement in parent-reported child symptoms (appetite, pain, headache, sleep, physical activity, fatigue). A staged nurse-led coaching program resulted in no changes to physical activity, and results showed a significant increase in fatigue at 6 months (39).

## Discussion

Compared to other cancer types, only a small number of studies have examined the effects of therapeutic exercise in pediatric survivors of solid tumors. Studies conducted to date vary considerably in methodological quality, exercise modalities, program duration, and clinical endpoints. The available evidence, although limited, supports the feasibility and safety of therapeutic exercise in this patient group, but well-designed trials are needed to assess the efficacy of such programs.

This review identified a small number of studies of mostly low methodological quality. Over a third (36%) of studies did not have a control group, and only five papers (29%) scored good-excellent quality on the PEDro Scale. Furthermore, over half of the studies (55%) evaluated small samples (*n* = <30), thus limiting statistical power. Brain cancer and other solid tumor diagnoses are relatively rare and the recruitment process is time- and resource-intensive, making it difficult to recruit the sample sizes required for adequately powered studies (51). Most studies did not include extended follow-up periods (>3 months) thus, the long-term effectiveness of the interventions, remains unclear. To address these methodological limitations, adequately powered multi-site studies employing rigorous study designs and long-term follow-up are needed. Alternative study design and analytic approaches also need to be explored to address limitations of small sample sizes (i.e., single-subject study designs) (52, 53).

The age of participants, tumor type, and time since diagnosis varied considerably across studies. Participants were, however, mostly “school-aged” (i.e., 5–18 years), highlighting an absence of studies involving young children aged 0 to 5 years. This is despite almost half (47%) of all childhood cancers diagnosed between the ages of 0 to 4 years (2). Early childhood is a crucial period of growth and development, where minimizing the impact of impairments, optimizing neuroplasticity, and enhancing rehabilitation is critical (54, 55). More research is needed to investigate the effects of therapeutic exercise in children with solid tumors during early childhood.

Children with brain cancer (e.g., medulloblastoma) were more frequently studied than other solid tumors, likely due the higher prevalence rates and high levels of treatment complications and long-term impairments (13). Challenges in crossing the blood-brain barrier mean that treatment for CNS tumors differ to that of other solid tumors, with brain cancer treated with more surgery and radiation-focused treatments compared to other solid tumors, which are more often treated with a rigorous regimen of surgery and chemotherapy (56, 57). Children with bone tumors in particular, (e.g., osteosarcoma) have their own unique experiences and needs, often effected by amputations, disability and poorer QoL (7, 58). Currently there is a near-absence of evidence investigating the role exercise may play in the survivorship phase of these children. As this group of children present different clinically, and undergo different treatment regimes, they may also respond differently to exercise. Regardless of the diagnoses, providing supportive care throughout the course of a child's acute treatment and survivorship is critical in achieving optimal outcomes.

The exercise programs varied by setting, level of supervision, length, and exercise modalities. Most studies delivered the exercise program in hospital-based settings, with few utilizing home-based programs. No studies delivered the exercise program in a community-based setting such as recreational halls, and parks. Whilst hospitals may be rich in resources (e.g., qualified staff, specific equipment), they also place additional challenges on children and their families. Hospitals can be inconvenient and expensive for travel, and for those children in the post-treatment phase, returning to hospital can induce fear and anxiety (59). The appropriate exercise setting is therefore crucial and may influence adherence, enjoyment, and long-term participation. To better meet the needs of children with solid tumors and their families, future research should investigate the effectiveness of patient-centered therapeutic exercise programs delivered in community-based settings.

Most programs included in this review involved face-to-face supervision by a trained exercise professional. Some patients and their families prefer flexible and convenient modes of intervention delivery (e.g., face-to-face at home, telehealth, mobile-health apps), which may enhance exercise adherence and long-term sustainability of program outcomes (59, 60). While supervised exercise may be initially required for monitoring safety and ensuring program fidelity, these remote delivery modes warrant investigation to delineate what is the most feasible and effective for children and their families who desire alternative delivery modes (e.g., children living in remote communities).

Studies in this review predominantly used adult-based exercise modalities and prescription closely aligned with generic American College of Sports Medicine guidelines (61). Few studies employed play- or game-based exercise, which is more likely to be engaging and motivating for children (62). Children are not little adults, and exercise programs for young children should be designed and implemented in a developmentally appropriate manner. Exercise interventions will likely be more effective and result in sustainable improvements in habitual physical activity, movement competence, and functional capacity if they are play- or game-based. Evaluations of developmentally appropriate play-based exercise programs in pediatric survivors of brain and other solid tumors are urgently needed to determine its effectiveness in this clinically unique patient group.

There was a notable lack of studies evaluating patient-centered, personalized exercise programs (63, 64), with multiple programs (*n* = 5) assessing traditional ‘impairment-based' exercise interventions (32–36, 40, 45–47, 50). Programs of this type are based on the expectation that remediating impairments will lead to improved participation in their activities of choice. However, such interventions typically report poor participation outcomes in pediatric cohorts (65, 66). Intrinsically-motivated behavior (e.g., regular physical activity) is theorized to be influenced by three basic psychological needs; autonomy, perceived competence, and relatedness (67, 68). Autonomy-supportive environments encourage intrinsic motivation for sustainable behavior change through fulfillment of these basic psychological needs (69, 70). An example of fostering an autonomy-supportive environment is goal setting, whereby goals are set collaboratively by the patients and their families to be meaningful, individualized, and sensitive to the patient's clinical presentation and preferences. There is evidence supporting the effectiveness of goal-directed interventions to improve motor performance, physical activity and QoL in other pediatric groups (e.g., children with cerebral palsy, muscular dystrophy, or intellectual disability), which may be more conducive to long-term improvements (71–73). There are a small number of published study protocols advocating for the use of goal-directed interventions (74–76).

Although some outcomes were assessed in multiple studies, the measures used to assess these outcomes varied considerably. This finding is consistent with the conclusions of recent reviews on assessment of physical function in pediatric cancer patients (77–79). Outcomes measures were predominantly impairment-focused and lacked participation-level outcomes, based on the ICF framework. To improve the consistency, comparability, and transparency of study findings, future studies should adopt a more standardized approach to outcome selection and reporting. Future studies should also include participation-based outcomes such as the Participation and Environment Measure for Children and Youth (80) or participation-focused goal-directed measures, including the Canadian Occupational Performance Measure (81) or the Goal Attainment Scale (82).

Considering the relatively small number of studies and the wide range of outcomes and measures utilized across studies, it was not feasible to conduct a quantitative synthesis of the results. Nevertheless, across studies, therapeutic exercise was associated with improvements in cardiorespiratory fitness, habitual physical activity, muscle strength, functional mobility, motor performance, body composition and QoL. There were no reported adverse events from participation in therapeutic exercise. This suggests that therapeutic exercise is a potentially safe and feasible intervention for improving the QoL and wellbeing in pediatric survivors of brain cancer and other solid tumors; however, higher grade evidence is needed to make firm conclusions about the effectiveness of therapeutic exercise in this patient group and formal clinical recommendations.

This review has several strengths. It is the first scoping review of exercise training studies conducted in pediatric survivors of brain cancer and other solid tumors. The review was completed according to the PRISMA_ScR guidelines. An extensive search for articles was conducted across four databases, with no limitation on publication date. To supplement this search, an extensive manual search of all included articles was conducted. The review and extraction processes were rigorous. Titles and abstracts and full -text citations were reviewed by two independent authors and all citations were independently checked for accuracy after extraction. Opposing these strengths were some limitations. It is possible that not all relevant publications were identified through the systematic search and cross-reference searches. For example, papers published in languages other than English or in the gray literature were not included in the review. Despite most participants being “school-aged”, a small of number of studies included participants who were 18 years or over.

## Conclusion

This scoping review provides the first synthesis on the extent and nature of research pertaining to therapeutic exercise programs conducted in pediatric survivors of brain cancer and other solid tumors. Compared to blood cancer types, a small number of studies have examined the effects of therapeutic exercise in pediatric survivors of solid tumors. The methodological quality of studies conducted to date has been low (i.e., non-randomized study designs, no control group, small sample sizes) and have limited follow-up (e.g., greater than 6 months). Most of the research has been conducted in brain cancer survivors, with the bulk of studies evaluating highly structured supervised exercise programs delivered in hospital settings. The role of therapeutic exercise in children with other solid tumors, such as osteosarcoma and Ewing's sarcomas, has received limited research attention, particularly in relation to QoL in survivorship. Few studies employed play- or game-based exercise programs, instead utilizing adult-based modalities and prescription. There were multiple measures used for each outcome, with a paucity of standardized outcomes measures administered across papers. Although limited, the extant research supports the feasibility and safety of therapeutic exercise for children with solid tumors before, during and after treatment. Nonetheless, significant knowledge gaps were identified. Future research should address the major gaps in the literature, including the evaluation of developmentally appropriate play-based physical activity interventions for children aged 5 years and under; the feasibility, acceptability, and potential efficacy of exercise programs delivered in of different settings (e.g., home and community-based settings) and delivery channels (e.g., telehealth and apps). To improve the quality of evidence, collaborative, multi-site studies are needed to ensure that trials are adequately powered to detect clinically meaningful changes in outcomes.

## Author contributions

ST, CS, EB, and BK were responsible for the conceptualization and design of the study. CS, EB, and BK screened citations and critically appraised included papers. BK completed data extraction and drafted the manuscript, which was critically reviewed by ST, CS, EB, and NB. All authors read and approved the final manuscript.

## Funding

This review is supported by a project grant from the Children's Brain Cancer Centre through the Children's Hospital Foundation (CCABCR010). The funders had no role in the design of the study or preparation of the manuscript, and will remain separate for data collection, analysis, and interpretation of the data.

## Conflict of interest

The authors declare that the research was conducted in the absence of any commercial or financial relationships that could be construed as a potential conflict of interest.

## Publisher's note

All claims expressed in this article are solely those of the authors and do not necessarily represent those of their affiliated organizations, or those of the publisher, the editors and the reviewers. Any product that may be evaluated in this article, or claim that may be made by its manufacturer, is not guaranteed or endorsed by the publisher.
