# Supplementary material for: Therapeutic exercise interventions in pediatric survivors of brain cancer and other solid tumors: A scoping review
Source: Front Pediatr. 2022 Sep 16;10:979292. doi: 10.3389/fped.2022.979292 (PMC9535626; doi:10.3389/fped.2022.979292)
Supplement: Supplementary file 1 [file Data_Sheet_1.pdf]

Supplementary Material 1

PubMed search strategy

(((Neoplasms[MeSH]) OR tumor*[Title/Abstract]) OR tumour*[Title/Abstract] OR cancer*[Title/Abstract] OR neoplasm*[Title/Abstract] OR sarcoma*[Title/Abstract] OR carcinoma*[Title/Abstract] OR glioma*[Title/Abstract] OR "acquired brain injury"[Title/Abstract] OR malignan*[Title/Abstract] OR oncol*[Title/Abstract]) AND (((pediatric*[Title/Abstract]) OR paediatric*[Title/Abstract]) OR child*[Title/Abstract] OR youth[Title/Abstract] OR adolescen*[Title/Abstract] OR AYA[Title/Abstract] OR teen*[Title/Abstract]) AND (((Exercise[MeSH]) OR Exercis*[Title/Abstract]) OR physiotherapy[Title/Abstract] OR "physical therapy"[Title/Abstract] OR "physical activit*"[Title/Abstract] OR "motor activity"[Title/Abstract] OR training[Title/Abstract] OR strength[Title/Abstract] OR resistance[Title/Abstract] OR dance[Title/Abstract] OR "games"[Title/Abstract] OR "virtual reality"[Title/Abstract] OR aerobic[Title/Abstract] OR fitness[Title/Abstract] OR walk*[Title/Abstract] OR movement[Title/Abstract] OR "functional ability"[Title/Abstract] OR "physical function"[Title/Abstract] OR "functional capacity"[Title/Abstract] OR flexibility[Title/Abstract] OR sport*[Title/Abstract] OR "physical performance"[Title/Abstract] OR "physical endurance"[Title/Abstract]) AND (((intervention[Title/Abstract]) OR program*[Title/Abstract]) OR trial[Title/Abstract] OR "pilot study"[Title/Abstract] OR "cohort study"[Title/Abstract] OR trail[Title/Abstract])
