# Supplementary material for: Therapeutic exercise interventions in pediatric survivors of brain cancer and other solid tumors: A scoping review
Source: Front Pediatr. 2022 Sep 16;10:979292. doi: 10.3389/fped.2022.979292 (PMC9535626; doi:10.3389/fped.2022.979292)
Supplement: Supplementary file 2 [file Table_1.pdf]

Supplementary Table 1: Characteristics and outcomes of included studies

Study and participant characteristics			Exercise training				Outcomes	
Author (year)<br>Country<br>Study design<br>PEDro score	Sample size n=, each group n =, age (years, mean ± SD), % female, diagnoses	Time since diagnosis (months, mean ± SD)	Timing of training, Duration, Group or individual program, Setting, Supervision	FITT<br>(F: Frequency; I: Intensity; T: Time; T: Type)	Control details	Adherence	Outcomes	Main findings
Fiuza-Luces et al. (2016)(40)<br><br>Spain<br><br>**RCT**<br><br>9/11	n = 49<br><br>*CG*: n = 25, 11 ± 1, 28%<br>*IG*: n = 24, 10 ± 1, 29%<br><br>Soft tissue sarcomas, bone tumors, neuroblastomas, Wilms tumors, germ cell tumors, Hodgkin and non-Hodgkin lymphoma	Not reported	• Neoadjuvant chemotherapy<br>• Approximately 19 weeks<br>• Individual<br>• Inpatient<br>• Supervised by experienced fitness professional	**F**: x 3/week<br>**I**: 60-70% heart rate max and x 8-15 repetitions x 2-3 sets, 1 to 2-minute rests<br>**T**: 60-70 minutes; 30 minutes strength and 30 minutes aerobic<br>**T**: cycle ergometers, active games, and resistance training for most major muscle groups	Regular hospital care, including physiotherapy as needed	68% of participants completed >90% of prescribed exercises	Strength (5RM), cardiorespiratory fitness (GXT), functional mobility (TUG, TUDS), physical activity (wearable), QoL (PedsQL)	↑ strength of leg press, bench press and lateral row*<br><br>Strength tended to decrease with detraining<br><br>Trend was found toward a training-induced improvement in ventilatory threshold
Fiuza-Luces et al. (2017)(47)<br><br>Spain<br><br>**RCT**<br><br>6/11	n = 49<br><br>*CG*: n = 25, 11 ± 1, 28%<br>*IG*: n = 24, 10 ± 1, 29%<br><br>Soft tissue sarcomas, bone tumors, neuroblastomas, Wilms tumors, germ cell tumors, Hodgkin and non-Hodgkin lymphoma	Not reported	• Neoadjuvant chemotherapy<br>• Approximately 19 weeks<br>• Individual<br>• Inpatient<br>• Supervised by experienced fitness professional	**F**: x 3/week<br>**I**: 60-70% heart rate max and x 8-15 repetitions x 2-3 sets, 1 to 2-minute rests<br>**T**: 60-70 minutes; 30 minutes strength and 30 minutes aerobic<br>**T**: cycle ergometers, active games, and resistance training for most major muscle groups	Regular hospital care, including physiotherapy as needed	68% of participants completed >90% of prescribed exercises	Immune cell counts, physical activity (wearable)	Trend was found toward an interaction effect for natural killer cells expressing the immunoglobulin-like receptor KIR2DS4

Author (year)<br>Country<br>Study design<br>PEDro score	Sample size n=, each group n =, age (years, mean ± SD), % female, diagnoses	Time since diagnosis (months, mean ± SD)	Timing of training, Duration, Group or individual program, Setting, Supervision	FITT<br>(F: Frequency; I: Intensity; T: Time; T: Type)	Control details	Adherence	Outcomes	Main findings
Sabel et al. (2016)(41)<br><br>Sweden<br><br>**RCT with crossover**<br><br>8/11	n = 13<br><br>*WG:* n = 6, 13.2 ± 1.9, 50%<br>*IG*: n = 7, 11.9 ± 3.6, 57%<br><br>Anaplastic astrocytoma, choroid plexus carcinoma, germ cell tumor, medulloblastoma, pilocytic astrocytoma, primitive neuroectodermal tumor	12 to 60	• 1-5 years post-treatment<br>• 10-12 weeks<br>• Individual<br>• Home<br>• Once weekly coaching sessions by a nurse	**F**: x 5/week<br>**I**: not reported<br>**T**: 30 minutes<br>**T**: active video gaming-games chosen by investigators	Waitlist group asked to avoid active videogaming. Otherwise, live their life as normal	100%- all participants wore monitors for five days/week as targeted	Physical activity (wearable), motor performance (BOT-2)	↓ of 14% in sedentary time during the first 12 days^<br><br>↑ body coordination score immediately post-intervention^<br><br>↑ manual coordination score immediately post-intervention
Sabel et al. (2017)(42)<br><br>Sweden<br><br>**RCT with crossover**<br><br>8/11	n = 13<br><br>*WG:* n = 6, 13.2 ± 1.9, 50%<br>*IG*: n = 7, 11.9 ± 3.6, 57%<br><br>Anaplastic astrocytoma, choroid plexus carcinoma, germ cell tumor, medulloblastoma, pilocytic astrocytoma, primitive neuroectodermal tumor	12 to 60	• 1-5 years post-treatment<br>• 10-12 weeks<br>• Individual<br>• Home<br>• Once weekly coaching sessions by a nurse	**F**: x 5/week<br>**I**: not reported<br>**T**: 30 minutes<br>**T**: active video gaming-games chosen by investigators	Waitlist group asked to avoid active videogaming. Otherwise, live their life as normal	100%- all participants wore monitors for five days/week as targeted	Cognitive tests, ADLs (AMPs)	↑ ADL motor skill score and process score immediately post-intervention^<br><br>ADL process skills improved to a score above the cut-off for independent living post-intervention#

Author (year)<br>Country<br>Study design<br>PEDro score	Sample size n=, each group n =, age (years, mean ± SD), % female, diagnoses	Time since diagnosis (months, mean ± SD)	Timing of training, Duration, Group or individual program, Setting, Supervision	FITT<br>(F: Frequency; I: Intensity; T: Time; T: Type)	Control details	Adherence	Outcomes	Main findings
Corr et al. (2017)(32)<br><br>United States<br><br>**non-RCT**<br><br>6/11	n = 49<br><br>*CG*: n = 35, 13.1 ± 3.5, 46%<br>*IG*: n = 14, 13.5 ± 3.5, 29%<br><br>Osteosarcoma, Ewing's sarcoma, chondroblastoma	Not reported	• Neoadjuvant chemotherapy<br>• 10-12 weeks<br>• Individual<br>• Pre-operative and post-operative as inpatient + outpatient<br>• Supervised by physiotherapist	**F**: x 3/week<br>**I**: 50-70% heart rate max and x10 repetitions x3 sets<br>**T**: 60 minutes<br>**T**: mobilising, cycle ergometry, Wii Sports, resistance training for most major muscle groups	No pre-operative intervention. Usual post-operative care	85% of participants completed >50% of scheduled PT sessions	Functional mobility (FMA, ROM), strength (MMT)	↑ functional mobility score at 20-22 weeks*<br><br>↑ in 9MWR at 20-22 weeks*
Müller et al. (2014)(45)<br><br>Germany<br><br>**non-RCT**<br><br>5/11	n = 21<br><br>*CG*: n = 11, 12.5 ± 2.6, 55%<br>*IG*: n = 10, 15.5 ± 2.0, 60%<br><br>Anaplastic ependymoma, medulloblastoma, atypical neurocytoma, glioma, CNS germ cell tumor, retinoblastoma, osteosarcoma and Ewing's sarcoma (all pelvis and lower limb)	*IG*: 3.6 ± 0.5<br><br>*CG*: 3.6 ± 0.5	• Neoadjuvant and adjuvant treatment inpatient stays<br>• Approximately 40 weeks<br>• Individual<br>• Inpatient<br>• Supervised by two sport scientists	**F**: x 3-4/week<br>**I**: BORG RPE rating 13-16 and x 6-12 repetitions x 1-3 sets<br>**T**: 15-45 minutes<br>**T**: treadmill, cycle ergometry, sports, balance (single leg stance), strength (cable row machine)	Usual care consisting of 20–30-minutes daily mobilisation techniques offered on weekdays only	77% total available sessions attended	Body composition (DXA), physical activity (wearable)	↓ reductions in bone mass were observed in the exercise group in all scanned regions, except for calcaneus on unaffected side<br><br>↑ step counts per day and minutes in MVPA at 6- and 12-months*

Author (year)<br>Country<br>Study design<br>PEDro score	Sample size n=, each group n =, age (years, mean ± SD), % female, diagnoses	Time since diagnosis (months, mean ± SD)	Timing of training, Duration, Group or individual program, Setting, Supervision	FITT<br>(F: Frequency; I: Intensity; T: Time; T: Type)	Control details	Adherence	Outcomes	Main findings
Spreafico et al. (2021)(46)<br><br>Italy<br><br>**non-RCT**<br><br>5/11	n = 44<br><br>*CG:* n = 23, 8-21 years, 48%<br>*IG:* n = 21, 5-26 years, 43%<br><br>Nasopharyngeal carcinoma, thyroid carcinoma, non-Hodgkin's lymphoma, brain tumor, neuroblastoma, soft tissue, sarcoma, Ewing's sarcoma, abdominal desmoplastic tumor, Wilms tumor	Not reported	• During or after treatment<br>• 6 weeks<br>• Individual<br>• Inpatient<br>• Supervised by a sports trainer	**F**: x 3/week<br>**I**: not reported<br>**T**: 60 minutes<br>**T**: aerobic (stationary bike or treadmill), strength (body weight and resistance bands), stretching	Not reported	Not reported	QoL (PedsQL), fatigue (PedsQL-MFS)	Non-significant improvements in emotional and social functioning in the intervention group<br><br>No significant change in overall QoL or fatigue scores
Hooke et al. (2019)(39)<br><br>United States<br><br>**non-RCT**<br><br>4/11	n = 57<br><br>*CG*: n = 27, 12.8 ± 3.3, 48%<br>*IG:* n = 30, 12 ± 3.6, 37%<br><br>Acute lymphoblastic leukemia, lymphoma, solid tumors	Not reported	• During and/or after treatment<br>• During routine clinic visits<br>• Individual<br>• Inpatient<br>• Coached by a nurse practitioner	Five-stage coaching program, consisting of: 1) asking about current PA, 2) assessing if the patient has health barriers to PA, 3) determining the stage of change the patient and the parent are in and implementing the action appropriate for the stage, 4) writing the PA prescription (prescription template used with CDC recommendations), 5) providing information on resources	Intervention described in this study is part of standard care. Historical group did not receive this intervention but received physical therapy as usual	Patient adherence not reported<br><br>16% of electronic medical records included documentation of intervention delivery	Physical activity (wearable and GLTEQ), fatigue (Childhood Fatigue Scale)	No changes to PA compared to the control group at 4 months<br><br>↑ fatigue scores in solid tumor subgroup[^]

Author (year)<br>Country<br>Study design<br>PEDro score	Sample size n=, each group n =, age (years, mean ± SD), % female, diagnoses	Time since diagnosis (months, mean ± SD)	Timing of training, Duration, Group or individual program, Setting, Supervision	FITT<br>(F: Frequency; I: Intensity; T: Time; T: Type)	Control details	Adherence	Outcomes	Main findings
Cox et al. (2020)(33)<br><br>Canada<br><br>**non-RCT with cross over**<br><br>4/11	n = 25<br><br>*WG:* n = 11, 12.5 ± 2.9, 50%<br>*IG*: n = 14, 11.1 ± 3.2, 36%<br><br>Hemispheric or posterior fossa brain tumors treated with craniospinal radiation	*WG*: 70.3 ± 41.2<br><br>*IG*: 63.4 ± 28.8	• 1-10 years post-treatment<br>• 12 weeks<br>• Individual and group-based<br>• Setting 1: Outpatient+ Home. Setting 2: Outpatient<br>• Supervised by physical therapist, kinesiologist or experienced fitness trainer	**F**: Setting 1- x 2 group sessions + x 2 home sessions/week<br>Setting 2- x 3 group sessions/week<br>**I**: 80% peak heart rate<br>**T**: 90-minute group sessions; 30-minute home sessions<br>**T**: running, relays, obstacle courses, dodgeball, basketball	Continue with normal routine	84% total available sessions attended	Brain function (response accuracy and latency, functional connectivity)	↑ theta, alpha and high gamma frequency band coherence following exercise training, compared to no exercise*<br><br>Significant carryover effect observed for response accuracy in Go/No-Go trials following exercise training^
Riggs et al. (2017)(34)<br><br>Canada<br><br>**non-RCT with cross over**<br><br>5/11	n = 28<br><br>*WG:* n = 12, 12.0 ± 3.0, 44%<br>*IG*: n = 16, 11.2 ± 3.0, 42%<br><br>Hemispheric or posterior fossa brain tumors	*WG*: 70.6 ± 40.1<br><br>*IG*: 66.4 ± 28.6	• 1-10 years post-treatment<br>• 12 weeks<br>• Individual and group-based<br>• Setting 1: Outpatient + Home. Setting 2: Outpatient<br>• Supervised by physical therapist, kinesiologist or fitness trainer	**F**: Setting 1- x 2 group sessions + x 2 home sessions/week<br>Setting 2- x 3 group sessions/week<br>**I**: 80% peak heart rate<br>**T**: 90-minute group sessions; 30-minute home sessions<br>**T**: running, relays, obstacle courses, dodgeball, basketball	Continue with normal routine	84% total available sessions attended	Brain structure (MRI), cognitive skills (CANTAB), cardiorespiratory fitness (6MWT)	↑ fractional anisotropy in cross corpus callosum, cingulum, and superior longitudinal fasciculi bilaterally and right corticospinal tract and inferior frontal occipital fasciculi, maintained at 12 weeks^<br><br>↑ hippocampal volume in group training setting only^ Maintained at 12 weeks<br><br>↓ reaction time in group setting only post-intervention and at 12 weeks^<br><br>↑ cardiorespiratory endurance across both training settings post-intervention^

Author (year)<br>Country<br>Study design<br>PEDro score	Sample size n=, each group n =, age (years, mean ± SD), % female, diagnoses	Time since diagnosis (months, mean ± SD)	Timing of training, Duration, Group or individual program, Setting, Supervision	FITT<br>(F: Frequency; I: Intensity; T: Time; T: Type)	Control details	Adherence	Outcomes	Main findings
Piscione et al. (2017)(35)<br><br>Canada<br><br>**non-RCT with cross over**<br><br>4/11	n = 28<br><br>*WG:* n = 12, 12.0 ± 3.0, 44%<br>*IG*: n = 16, 11.2 ± 3.0, 42%<br><br>Hemispheric or posterior fossa brain tumors	*WG*: 70.6 ± 40.1<br><br>*IG*: 66.4 ± 28.6	• 1-10 years post-treatment<br>• 12 weeks<br>• Individual and group-based<br>• Setting 1: Outpatient+ Home. Setting 2: Outpatient<br>• Supervised by physical therapist, kinesiologist or fitness trainer	**F**: Setting 1- x 2 group sessions + x 2 home sessions/week<br>Setting 2- x 3 group sessions/week<br>**I**: 80% peak heart rate<br>**T**: 90-minute group sessions; 30-minute home sessions<br>**T**: running, relays, obstacle courses, dodgeball, basketball	Continue with normal routine	84% total available sessions attended	Motor performance (BOT-2), cardiorespiratory fitness (GXT)	↑ bilateral coordination despite training condition, with carryover effect to 12 weeks^<br><br>Clinically meaningful improvement in bilateral coordination between groups#<br><br>↑ pro-rated work rate in group training setting only, immediately following intervention and at 12 weeks^
Szulc-Lerch et al. (2018)(36)<br><br>Canada<br><br>**non-RCT with cross over**<br><br>4/11	n = 28<br><br>*WG:* n = 12, 12.0 ± 3.0, 44%<br>*IG*: n = 16, 11.2 ± 3.0, 42%<br><br>Hemispheric or posterior fossa brain tumors	*WG*: 70.6 ± 40.1<br><br>*IG*: 66.4 ± 28.6	• 1-10 years post-treatment<br>• 12 weeks<br>• Individual and group-based<br>• Setting 1: Outpatient+ Home. Setting 2: Outpatient<br>• Supervised by physical therapist, kinesiologist or fitness trainer	**F**: Setting 1- x 2 group sessions + x 2 home sessions/week<br>Setting 2- x 3 group sessions/week<br>**I**: 80% peak heart rate<br>**T**: 90-minute group sessions; 30-minute home sessions<br>**T**: running, relays, obstacle courses, dodgeball, basketball	Continue with normal routine	84% total available sessions attended	Brain structure (MRI)	↑ cortical thickness in right pre and postcentral gyri, left temporal pole, left superior temporal gyrus and left parahippocampal gyrus immediately post-intervention in group setting only^<br><br>↑ white matter volume underlying the right motor and somatosensory cortices following exercise training^

Author (year)<br>Country<br>Study design<br>PEDro score	Sample size n=, each group n =, age (years, mean ± SD), % female, diagnoses	Time since diagnosis (months, mean ± SD)	Timing of training, Duration, Group or individual program, Setting, Supervision	FITT<br>(F: Frequency; I: Intensity; T: Time; T: Type)	Control details	Adherence	Outcomes	Main findings
Govardhan et al. (2019)(48)<br><br>India<br><br>**Single-arm pre-test post-test**<br><br>4/11	n = 18, 9.8, 50%<br><br>Anaplastic ependymoma, medulloblastoma, atypical neurocytoma, glioma, CNS germ cell tumor, retinoblastoma	Not reported	• Adjuvant treatment<br>• 4 weeks<br>• Individual<br>• Inpatient<br>• Supervised by trained instructor	**F**: x 3/week<br>**I**: not reported<br>**T**: 60 minutes<br>**T**: yoga (breathing, postures, relaxation and meditation)	Not applicable	100%. All participants completed minimum sessions (12 sessions over 4 weeks)	Parent-proxy report child treatment symptoms	Less pain, relief in headache, increase in appetite, better sleep, increase daily activity and reduced fatigue^
Sparrow et al. (2017)(38)<br><br>United States<br><br>**Single-arm pre-test post-test**<br><br>4/11	n = 9, 7.3 ± 3.6, 66%<br><br>Juvenile pilocytic astrocytoma, anaplastic astrocytoma, atypical teratoid rhabdoid tumor, high-grade infantile glioneuronal tumor, choroid plexus carcinoma, high-grade glioma. All participants experienced hemiplegia	50.4 ± 36.0	• Post-treatment<br>• 3 weeks<br>• Individual<br>• Outpatient + Home<br>• Supervised by a clinician trained who was trained through the University of Alabama Constraint Induced Therapy Pediatric Training Program	**F**: 180min x 5/week<br><br>Constraint-induced movement therapy (long arm removable cast). Cast remained in situ until 2 days before end of intervention, when bimanual training occurred. Home-based component: individualized home program that included activities and exercises to facilitate maintenance of gained skills. Weekly phone calls to check in	Not applicable	All participants completed all 15 sessions	Upper limb function (PMAL, PAFT, INMAP), QoL (PedsQL)	↑ amount and quality of affect arm use immediately post-intervention and at 3-months^ (except frequency of use at 3 months)<br><br>↑ parent-reported real-world function in amount, number of patterns, daily living skills and quality of affected arm use post-intervention and at 3-months^ (except amount)

Author (year)<br>Country<br>Study design<br>PEDro score	Sample size n=, each group n =, age (years, mean ± SD), % female, diagnoses	Time since diagnosis (months, mean ± SD)	Timing of training, Duration, Group or individual program, Setting, Supervision	FITT<br>(F: Frequency; I: Intensity; T: Time; T: Type)	Control details	Adherence	Outcomes	Main findings
Müller et al. (2016)(43)<br><br>Germany<br><br>**Single-arm pre-test post-test**<br><br>3/11	n = 64<br><br>*BT*: n = 38, 10.1 ± 3.5, 55%<br>SC: n = 26, 13.4 ± 3.5, 65%<br><br>Brain tumors and sarcomas	*BT*: 31.8 ± 25.3<br><br>*SC*: 26.3 ± 30.4	• Post-treatment<br>• 4 weeks<br>• Individual and group-based<br>• Inpatient<br>• One-hour initial consult with physiotherapist. Activity programs led by instructors or physiotherapists	**F**: approximately x 11/week<br>**I**: not reported<br>**T**: exercise therapy groups (30-60 minute), aquatic and hippotherapy (20 minutes) and land-based physiotherapy (30 minutes)<br>**T**: camp. Individual (land-based physiotherapy, aquatic exercises, hippotherapy) and group (exercise training and sports games)	Not applicable	Not reported	Physical activity (wearable), HRQoL (KINDL)	↑ gait cycles per day, gait cycles per hour and minutes in MVPA at 12 months post-intervention^<br><br>↑ peak 10-minute cadence immediately following intervention and at 12 months post-intervention^<br><br>↑ HRQoL for BT immediately post-intervention and at 6 months<br><br>↓ HRQoL well-being score for SC at 12 months^
Müller et al. (2017)(44)<br><br>Germany<br><br>**Single-arm pre-test post-test**<br><br>3/11	n = 88<br><br>*BT*: n = 59, 10.1 ± 3.8, 54%<br>SC: n = 29, 13.4 ± 3.6, 52%<br><br>Brain tumors and sarcomas	*BT*: 31.8 ± 25.3<br><br>*SC*: 26.3 ± 30.4	• Post-treatment<br>• 4 weeks<br>• Individual and group-based<br>• Inpatient<br>• One-hour initial consult with physiotherapist. Activity programs led by instructors or physiotherapists	**F**: approximately x 11/week<br>**I**: not reported<br>**T**: exercise therapy groups (30-60 minute), aquatic and hippotherapy (20 minutes) and land-based physiotherapy (30 minutes)<br>**T**: Individual (land-based physiotherapy, aquatic exercises, hippotherapy) and group (exercise training and sports games)	Not applicable	Not reported	Static balance (balance plate), gait quality (TEMPLO)	↑ mean and median single leg stance time following the intervention^<br><br>↓ mean vCoP following the intervention^<br><br>Moderate effect on walk ratio in impaired balance group^<br><br>↑ mean maximum knee flexion angle in gait following the intervention^

Author (year)<br>Country<br>Study design<br>PEDro score	Sample size n=, each group n =, age (years, mean ± SD), % female, diagnoses	Time since diagnosis (months, mean ± SD)	Timing of training, Duration, Group or individual program, Setting, Supervision	FITT<br>(F: Frequency; I: Intensity; T: Time; T: Type)	Control details	Adherence	Outcomes	Main findings
Ovans et al. (2018)(37)<br><br>United States<br><br>**Single-arm pre-test post-test**<br><br>2/11	n = 15, 11.5 ± 3.3, 33%<br><br>Astrocytoma, medulloblastoma, primitive neurodectermal tumor, craniopharyngioma, germ cell tumor	11.25 ± 8.1	• <2 years post-treatment<br>• 12 weeks<br>• Individual<br>• Home<br>• Five physiotherapy coaching sessions (every 2-3 weeks)	**F**: not reported<br>**I**: individualized to each child's current level of function, motivation to move and opportunities to increase steps<br>**T**: not reported<br>**T**: aerobic (daily step count goals + coaching on activities will help reach these goals)	Not applicable	All participants received 5 coaching sessions. Met or exceeded goal on 68.9% of weeks	Physical activity (wearable and GLTEQ), cardiorespiratory fitness (6MWT), QoL (PedsQL)	↑ 6MWT distance from baseline to 12 and 24 weeks^<br><br>↑ PedsQL physical health subscale score at 24 weeks^<br><br>↑ total fatigue scores from baseline to 12 and 24 weeks^

*statistically significant between-group comparison, ^statistically significant within-group comparison, #clinically meaningful difference

Definitions: ADLs: activities of daily living; AMPS: Assessment of Motor and Process Skills; BOT-2: Bruininks-Osterestsky Test of Motor Performance; BT: brain tumor; CANTB: Cambridge Neuropsychological Test Automated; CG: Control group; CNS: central nervous system; DXA: Dual-energy X-ray absorptiometry; FMA: Functional Mobility Assessment; GLTEQ: Godin Leisure-Time Exercise Questionnaire; GXT: graded exercise test; HRQoL: health-related quality of life; IG: Intervention group; INMAP: Inventory of New Motor Activities and Programs; KINDL: Kinder Lebensqualität Questionnaire; MFS: multidimensional fatigue scale; MMT: manual muscle test; MRI: magnetic resonance imaging; MVPA: moderate-to-vigorous physical activity; PAFT: Pediatric Arm Function Test; PMAL: Pediatric Motor Activity Log; QoL: quality of life; RCT: randomized controlled trial; ROM: range of motion; SC: sarcoma; SD: standard deviation; TUG: timed up and go; TUDS: timed up and down stairs; vCoP: velocity of the centre of pressure; WG: waitlist group; 5RM: five-repetition maximum; 6MWT: six-minute walk test; 9MWR: 9-minute walk-run test.
